# Disilicate Dental Ceramic Surface Preparation by 1070 nm Fiber Laser: Thermal and Ultrastructural Analysis

**DOI:** 10.3390/bioengineering5010010

**Published:** 2018-01-31

**Authors:** Carlo Fornaini, Federica Poli, Elisabetta Merigo, Nathalie Brulat-Bouchard, Ahmed El Gamal, Jean-Paul Rocca, Stefano Selleri, Annamaria Cucinotta

**Affiliations:** 1 Department of Engineering and Architecture, University of Parma, Parco Area delle Scienze 181/A, 43124 Parma, Italy; federica.poli@unipr.it (F.P.); stefano.selleri@unipr.it (S.S.); annamaria.cucinotta@unipr.it (A.C.); 2 Micoralis Laboratory, Faculty of Dentistry, University of Cote d’Azur, 24 Avenue des Diables Bleus, 06357 Nice, France; elisabetta.merigo@gmail.com (E.M.); Nathalie.BRULAT@unice.fr (N.B.-B.); Ahmed.ELGAMAL@unice.fr (A.E.G.); Jean-Paul.ROCCA@unice.fr (J.-P.R.)

**Keywords:** disilicate ceramics, fiber lasers, fiber Bragg grating, energy dispersive X-ray spectroscopy

## Abstract

Lithium disilicate dental ceramic bonding, realized by using different resins, is strictly dependent on micro-mechanical retention and chemical adhesion. The aim of this in vitro study was to investigate the capability of a 1070 nm fiber laser for their surface treatment. Samples were irradiated by a pulsed fiber laser at 1070 nm with different parameters (peak power of 5, 7.5 and 10 kW, repetition rate (RR) 20 kHz, speed of 10 and 50 mm/s, and total energy density from 1.3 to 27 kW/cm^2^) and the thermal elevation during the experiment was recorded by a fiber Bragg grating (FBG) temperature sensor. Subsequently, the surface modifications were analyzed by optical microscope, scanning electron microscope (SEM), and energy dispersive X-ray spectroscopy (EDS). With a peak power of 5 kW, RR of 20 kHz, and speed of 50 mm/s, the microscopic observation of the irradiated surface showed increased roughness with small areas of melting and carbonization. EDS analysis revealed that, with these parameters, there are no evident differences between laser-processed samples and controls. Thermal elevation during laser irradiation ranged between 5 °C and 9 °C. A 1070 nm fiber laser can be considered as a good device to increase the adhesion of lithium disilicate ceramics when optimum parameters are considered.

## 1. Introduction

The demand for ceramic prosthetic restorations has become increasingly common in daily dentistry. Moreover, the continuous need for an increased level of precision, particularly in cosmetic dentistry, where new materials—such as feldspathic ceramics—play an important role in prosthetic rehabilitations, is considered crucially important. Unfortunately, failure resulting from porcelain fracture has been reported as ranging from 2.3 to 8% [1]. Nevertheless, it seems to be a function of a multi-factorial reason [2,3,4], with the key cause attributed to the composite resin adhesion with porcelain. Therefore, it is necessary to condition the ceramic surface, which is considered very interesting [5,6].

The inside surface of the ceramic prosthetics must be conditioned for optimized micro-mechanical retention by the resin penetration into the ceramic micro-roughness; this treatment enhances the mechanical retention of cement by enlarging the surface in contact with the tooth structure through the creation of micro-porosities [7,8]. For producing surface roughness and for promoting micro-mechanical retention, different treatment methods, such as diamond roughening, air-particle abrasion with aluminium oxide, and acid etching have been proposed in the literature [7,8]. All these techniques have been investigated under in vitro conditions [9,10,11].

The use of laser technology for surface treatment has already been successfully applied in many industrial applications by the utilization of high power sources. Today, this technology represents a controllable and flexible technique for the modification of surface properties for different various materials [12,13], since laser parameters have the capability to influence and alter the surface microstructure [14]. The in vitro study reported here has the aim to verify the possibility of performing the surface treatment of Lithium disilicate ceramic specimens by the irradiation of a 1070 nm pulsed fiber laser. 

Fiber lasers act as sources where the active medium is an optical fiber with a core doped with active ions, such as Nd (neodymium), Yb (ytterbium), Er (erbium), or Tm (thulium) [15]. Fiber lasers differ from traditional solid-state lasers mainly by the form of the gain medium: in fact, bulk crystal lasers are typically based on conventional rod or slab geometries while, in the case of fiber lasers, active ions are added into the core of an optical fiber, often with a length of many meters [16]. These lasers operate in continuous wave (CW) or pulsed mode and emit in a wide range of wavelengths, which is a function of the dopants and host materials. CW output powers of several kW [17] and pulse energies up to around 30 mJ [18,19] can be currently obtained with Yb-doped fiber lasers.

## 2. Materials and Methods

The circular faces of twelve cylinders of lithium disilicate ceramics (e.max Press, Ivoclar, Bolzano, Italy) with a 10 mm diameter and an 8 mm length were processed into three 3 × 3 mm square zones by using a 1070 nm pulsed fiber laser (AREX 20, Datalogic, Bologna, Italy). This source has a maximum average output power of 20 W and a fixed pulse duration of 100 ns, thus providing a maximum peak power of 10 kW for a repetition rate of 20 kHz. 

The chemical composition of the ceramic used in the study, as described by the company, consists of SiO_2_ as the primary component (57–80%) and Li_2_O (11–19%), K_2_O (0–13%), P_2_O_5_ (0–11%), ZrO_2_ (0–8%), ZnO (0–8%) as additional components.

**Figure 1:**
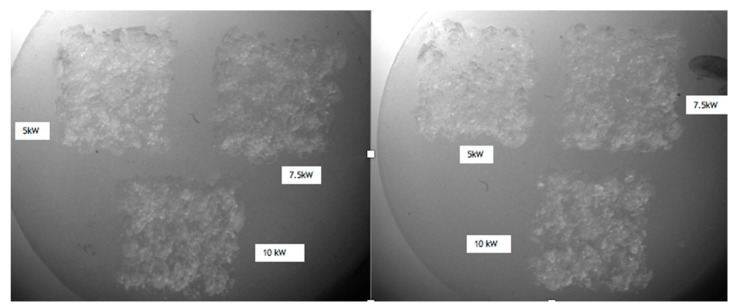
(**Left**) A Group A sample irradiated at 10 mm/s; (**Right**) A Group B sample irradiated at 50 mm/s.

After a preliminary pilot study, performed by optical microscope to evaluate the effects of different parameters, we used a repetition rate (RR) of 20 kHz; output powers of 5, 7.5 and 10 kW; and speeds of 10 and 50 mm/s. Thus, the cylinders were divided in two groups: six samples were irradiated at a speed of 10 mm/s (group A) and six at 50 mm/s (group B). On each sample of the two groups three zones were prepared at 5, 7.5 and 10 kW, respectively (Figure 1). As control group, a zone of the cylinders not irradiated by laser device was analyzed.

The lens used with the AREX 20 laser has a focal length of 160 mm. In this configuration, the laser beam has a spot-size of 80 μm. Each square zone on the sample surface has been processed using a meshed filling pattern with a distance between lines of 0.03 mm. The laser beam focalization was checked by a metal cylinder of the same dimension of the samples. The power per unit area deposited on the material ranged between 1.3 and 27 kW/cm^2^. The specimens were subsequently observed by an optical microscope (Olympus MTV-3, Tokyo, Japan), then metallized (ion sputter, Jeol JFC 1100E, Peabody, MA, USA) and analyzed by a Scanning Electron Microscope (SEM) (JSM-35CF, Jeol Ltd., Tokyo, Japan) and energy-dispersive X-ray spectroscopy (EDS) system (JED 2300F, Jeol Europe, Croissy-sur-Seine, France)

During the irradiation of the sample with the best laser parameters, the thermal elevation was recorded by a fiber Bragg grating (FBG)-based temperature sensor connected to an interrogator. The fiber sensor was positioned into the groove in the middle of the sample. A sm130-500 dynamic optical sensing interrogator (Micron Optics Inc., Atlanta, GA, USA) was used to measure the FBG wavelength shift induced by the temperature increase). This device is also considered as a compact, industrial-grade, dynamic optical sensor interrogation module, field-proven for robust, reliable, and long-term operation. The software included with the sensing interrogator system provides a single suite of tools for data acquisition, computation, and analysis of optical sensor networks. A 25 mm-long FBG with a center wavelength of 1550 nm, a reflectivity of 96%, and acrylate coating, imprinted in a standard SMF (AOS GmbH, Dresden, Germany), has been connected to the interrogator for performing the temperature change measurement. A temperature-induced wavelength shift of about 13 pm/°C has been considered for the FBG at 1550 nm.

The temperature increase during the laser irradiation has been measured only when the source operates with the best parameters, as per the observation of SEM and EDS analysis. The aim of this measurement was to provide the maximum value of the temperature rise, induced by the laser, that the disilicate ceramic material can withstand without being damaged. Higher-energy laser treatments provide a more significant temperature change, which is associated with the detrimental surface modifications, as shown by SEM and EDS analysis. With the FBG sensor it was also possible to see the time of the thermal elevation, also after the laser irradiation, until it decreased completely. The thermal elevation of the sample during the irradiation with the laser operating at a peak power of 5 kW, repetition rate of 20 kHz, and speed of 50 mm/s, has been recorded with a FBG connected to an interrogator.

## 3. Results

### 3.1. SEM Observation

**Figure 2:**
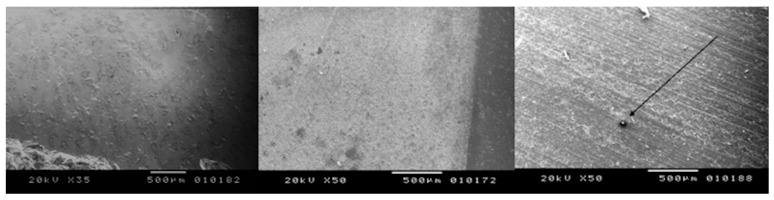
(**Left**) Non-irradiated sample; (**centre**) Peak power of 7.5 kW and 50 mm/s speed; (**right**) Peak power of 7.5 kW and 10 mm/s speed with a carbonization spot ((**left**) X35; (**centre**,**right**): X50).

By comparing the control group (non-irradiated samples) to the cylinders processed by the fiber laser at higher magnification, a greater difference can be noticed (Figure 2). 

**Figure 3:**
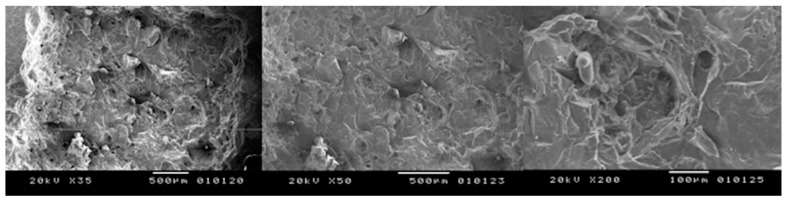
Peak power of 10 kW, speed of 10 mm/s: many zones with melting and carbonization are shown (**left**) X35; (**centre**) X200; (**right**) X500.

**Figure 4:**
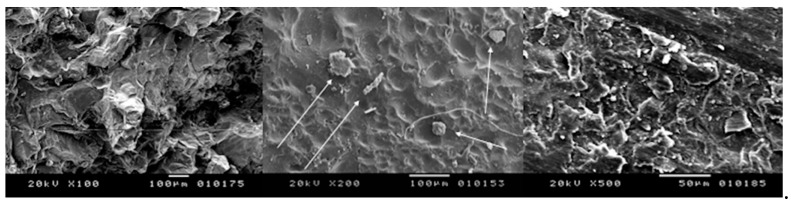
Peak power of 10 kW, speed of 50 mm/s: some points with melting are shown (**left**) X100; (**centre**) X200; (**right**) X500.

**Figure 5:**
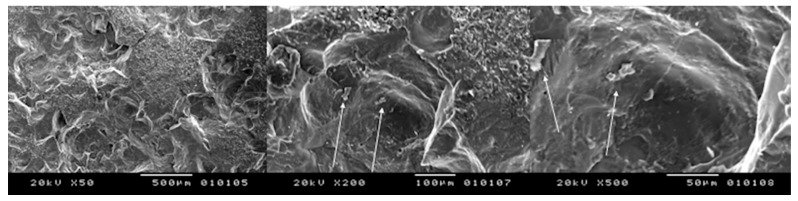
Peak power of 7.5 kW, speed of 50 mm/s: presence of melting and carbonization in some areas of the sample (**left**) X35; (**centre**) X50; (**right**) X200.

**Figure 6:**
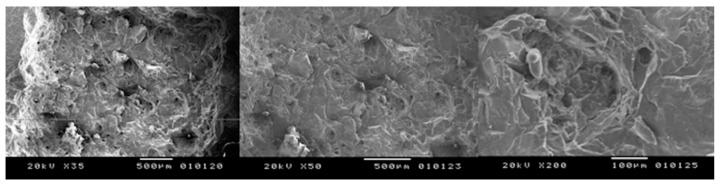
Peak power of 5 kW, speed of 10 mm/s: evidence of some zones with melting (**left**) X50; (**centre**) X100; (**right**) X500.

**Figure 7:**
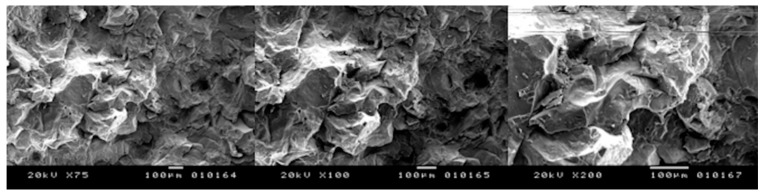
Peak power of 5 kW, speed of 50 mm/s: no evidence of carbonization and melting zones (**left**) X75; (**centre**) X100; (**right**) X200.

In fact, all the treated surfaces show a rough surface with many holes and irregularities. It is evident that the samples irradiated at different lasing parameters experienced some areas of melting and burning when the highest energy level was used, due to the cumulative effect of the laser energy. The presence of some cracks with variable intensities are also found due to the thermal effects of laser irradiation (Figure 3, Figure 4, Figure 5 and Figure 6). Only the samples of Group B irradiated at 5 kW did not show any thermal damages (Figure 7).

### 3.2. EDS Analysis

The EDS analysis consists of the percentage recording of chemical elements in the point where the probe is placed. Analyzed samples showed, in general, slight differences in the chemical composition between control groups and irradiated samples, even smaller variations by changing the lasing parameters were detected, thus confirming the information given by the SEM observation. 

**Figure 8:**
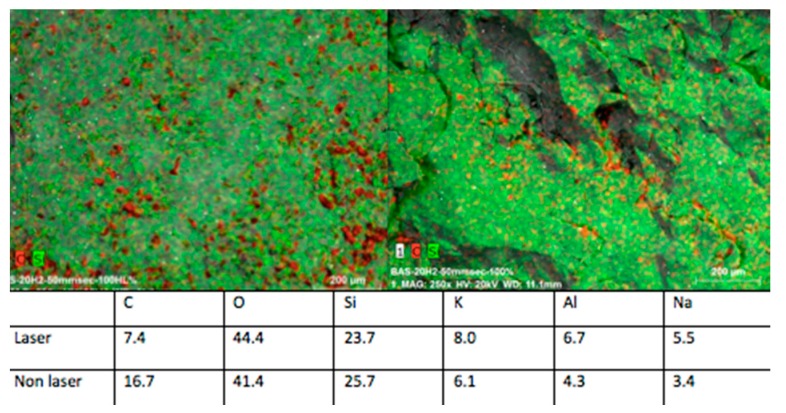
(**Left**) Control group and (**right**) samples irradiated with peak power of 10 kW, and a speed of 50 mm/s: the C concentration is shown in red.

The differences of elemental composition between the non-irradiated areas in the different samples may be explained by the structure of the ceramic, which is not homogeneous, thus resulting in structural variations of the tested zones (Figure 1, left). The samples treated with the laser operating at a peak power of 10 kW, repetition rate of 20 kHz, and speed of 50 mm/s experienced some zones (red spots) of lower percentage of C when compared to the control group. On the other hand, O and Al elements were slightly higher in the affected zones (Figure 8).

**Figure 9:**
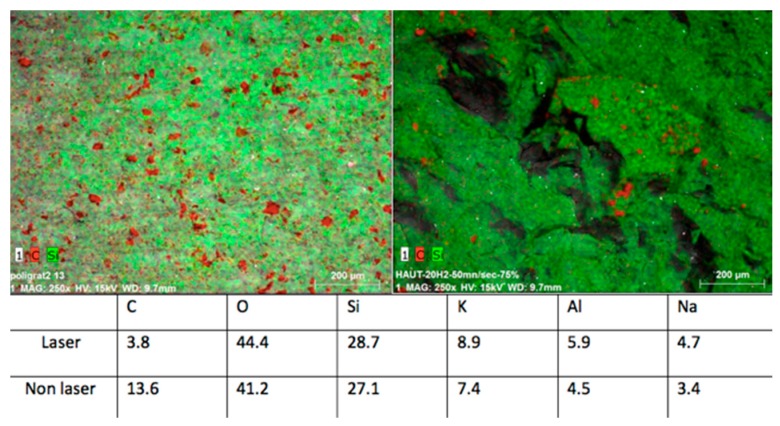
(**Left**) Control group and (**right**) samples irradiated with peak power of 7.5 kW and speed of 50 mm/s: the C concentration is shown in red.

The samples irradiated with a peak power of 7.5 kW, repetition rate of 20 kHz, and speed of 50 mm/s showed that only the carbon concentration was higher in the control group (13.6%), while all the other elements—such as O, Si, K, Al, and Na—presented higher concentration values on the treated surfaces (Figure 9).

**Figure 10:**
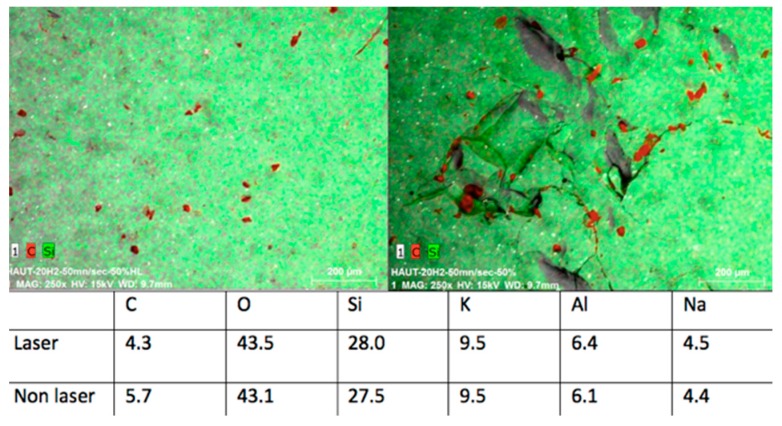
(**Left**) Control group and (**right**) samples irradiated with peak power of 5 kW and a speed of 50 mm/s: the C concentration is shown in red.

These data demonstrated a poor modification of the ceramic chemical structure caused by the laser operating with the optimum parameters (Figure 10).

### 3.3. Thermal Analysis

**Figure 11:**
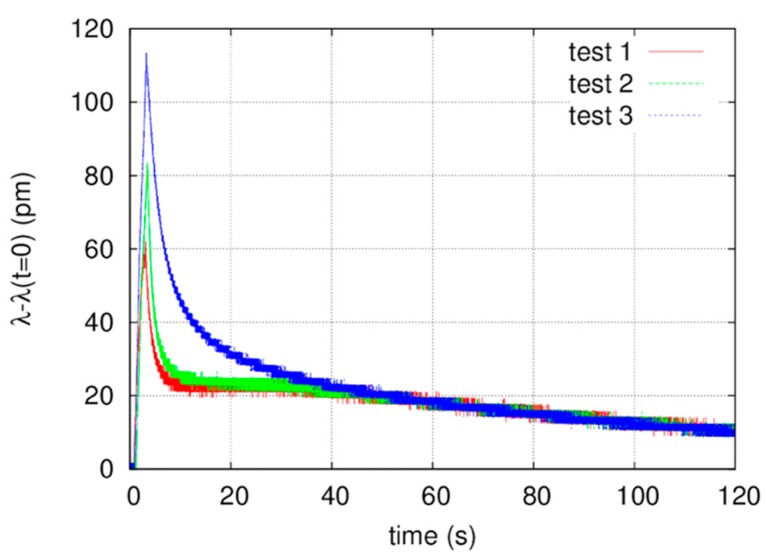
FBG sensor wavelength shift induced by temperature variations during and after the laser irradiation with the best parameters (peak power of 5 kW, repetition rate of 20 kHz, and speed of 50 mm/s).

The FBG wavelength shift obtained in a time interval of 120 s, during the laser processing, is reported in Figure 11. The temperature measurement has been repeated three times by processing three square regions on the sample surface. The fiber sensor was placed in the center of the sample, approximately at the same distance from all the areas irradiated by the laser. Notice that the wavelength shift measured by the interrogator is between 65 pm and 115 pm, respectively, in the first and the third test. Consequently, the temperature rise due to the laser processing is between 5 °C and 9 °C. The slight growth of the temperature value measured in the second and the third test may be due to the gradual heating of the sample, originating from the previous laser processing. Moreover, slight differences in the distance of the three zones irradiated by the laser with respect to the sensitive part of the fiber sensor must be taken into consideration.

The measure of the temperature rise during laser irradiation may shed some light on the explanation behind the crack formations. After laser irradiation, which could be explained through the high thermal effects of laser processing, along with the consequence of an extreme physical stress in the re-hardening ceramic surface. The importance of the very short pulse duration given by the fiber laser used in this study (100 ns) must also be underlined, which may explain the greater difference between the fluences of these tests, compared to those given in the cited works where irradiation had been performed in CW or in µs.

## 4. Discussion

The current adhesion strategies using resin bonding for ceramics involve micromechanical interlocking and chemical adhesive bonding and, thus, the ceramic surface requires surface roughening for mechanical bonding and surface activation for chemical adhesion. The search for non-destructive methods to treat inert ceramics for modifying their surface texture and chemical properties help to produce an activated/functionalized surface [20]. Hydrofluoric acid etching changes the surface of the glass ceramic by dissolving its glassy phase [21], and this process creates irregularities on the surface and increases the contact area between the adhesive system and the glass ceramic. 

The adhesion between dental ceramics and resin-based composites is the result of a physicochemical interaction across the interface between the resin (adhesive) and the ceramic (substrate). The physical contribution to the adhesion process is dependent on the surface treatment and topography of the substrate and can be characterized by its surface energy. Alteration of the surface topography by etching or airborne particle abrasion results in changes on the surface area and on the wettability of the substrate, which are related to the surface energy and the adhesive potential [22]. A rough surface increases the mechanical retention by enabling the adhesive to interlock with the surface irregularities created by the different conditioning methods [23]. Unfortunately, several studies demonstrated the possibility, by hydrofluoric acid, to damage the ceramic structure with the result increasing the fracture of the restorations [24].

Even the utilization of laser technology, recently introduced for this kind of treatment, is not free of problems. Particularly, some tests conducted on lithium disilicate [25] and CAD-CAM ceramics [26] with a CW CO_2_ laser at 10.6 μm confirmed the presence of micro-cracks and melting textures, due to the thermal effect of the laser irradiation at output powers higher than 10 W CW (3184.7 W/cm^2^). Moreover, the observation of the ceramics structure irradiated by a 10 W (14,185 W/cm^2^) pulsed Nd:YAP laser at 1340 nm exhibited the presence of holes, micro-cracks, and melted grains [25,26]. This is probably caused by the effect of a high quantity of radiation energy given in a well-defined portion of the ceramic surface over a short period, thus leading to a very high energy density accumulation. Micro-crack formation on ceramics after CO_2_ and Nd:YAP laser irradiations may be related to the high thermal effects of laser processing which leads to an extreme physical stress in the re-hardening ceramic surface [27,28]. Additionally, an Er:YAG laser was used for surface treatment of feldspathic porcelain, however, its effect resulted in a significantly weaker surface than that of the HF-treated surface. The probable assumption is that the laser energy from an Er:YAG laser is not well absorbed by the porcelain and, therefore, not sufficient to create a micro-mechanical retention pattern for more favorable bonding [29]. In agreement with this study, some authors affirmed that, even at a very high energy (500 mJ), an Er:YAG laser is not able to cause on the porcelain surface to roughen sufficiently to promote reliable adhesion to the resin composite [30]. Recently, the so-called “ultra-short pulses” lit up a greater interest in the field of the mean roughness value [31]. However, due to the higher expense associated with this laser source, to date, it is still utilized in only a few laboratories.

This study, based on the utilization of a 1070 nm pulsed fiber laser, demonstrated the ability, with the described optimal parameters, to characterize the ceramic surface for enhancing its adhesion to the enamel and dentine avoiding, at the same time, the thermal damages in the structure evidenced with the utilization of the other different wavelengths before described. 

The most common applications of fiber lasers regard the industrial field, where they are used mainly for material processing (i.e., for cutting and marking). The main utilizations of fiber lasers in medicine are related to lithotripsy [32], surgical treatment of vascular lesions [33], non-surgical skin aesthetic procedures [34,35], and eye surgery [36]. Recently, its use in the dental field also started to be considered, particularly in the surgery of soft oral tissues, where it demonstrated some advantages consisting of the scant overheating of the target and, consequently, scant tissue damages, probably also due to the shorter pulse duration (ns), compared to the emission normally used in dentistry (µs) [37].

This is also the reason for the great differences in the power densities utilized in this study (1.3 to 27 kW/cm^2^), compared to those used in the similar cited works [25,26] performed with different wavelengths. The measure of temperature rise during laser irradiation may shed some light on the explanation behind the crack formation after laser irradiation, which could be explained through the high thermal effects of laser processing, along with the consequence of an extreme physical stress in the re-hardening ceramic surface.

In fact, main process parameters in the laser–material interaction involve laser pulse duration and, consequently, it significantly affects the quality of the produced micro-feature and the material removal rate. 

Ablation of material can be facilitated by using short pulses as the laser energy is confined in a thin layer. For longer pulses, absorbed energy will be dissipated into the surrounding material by thermal processes. Absorption of long laser pulses also causes melting and substantial sputter evaporation of the material. These phenomena can contaminate the surrounding area, produce micro-cracks, and remove material over dimensions much larger than the laser spot. Other adverse effects include damage to adjacent structures, delamination, formation of recast material, and formation of a large heat affected zone (HAZ) [38]. This is also the reason for the low and short thermal elevation recorded by FBG during the irradiation of our samples, having a peak (5 to 9°) and coming back to the normality in 8 to 38 s as reported in Figure 10. 

It must also be underlined once more that the importance of the very short pulse duration given by the fiber laser used in this study (100 ns), which may explain the greater difference between the fluences of these tests, compared to those given in the cited works where irradiation had been performed in CW or in µs.

In this research, the laser parameters which seem to be the most effective for surface conditioning of the materials without causing any damages are a peak power of 5 kW, a repetition rate of 20 kHz, and a speed of 50 mm/s. In fact, the samples irradiated with these parameters revealed a rough surface with holes, irregularities, cavities, and recesses, while the presence of thermal damaging effects—such as melting, burning, and cracks—was not evident.

EDS observations of the samples irradiated with these parameters confirmed the SEM observation. The analysis, in this case, was conducted in four different zones and the results showed slight differences for all the elements concentrated in each analyzed zone, except for the decreasing of the C concentration in the irradiated samples, which is a result present also in other similar studies performed by different kinds of treatment, both thermal and non-thermal [25,39].

This study demonstrated that, by the proper parameters (peak power of 5 kW, repetition rate of 20 kHz, and speed of 50 mm/s), a 1070 nm pulsed fiber laser may characterize the surface of lithium disilicate ceramics to enhance their resin adhesion, with a low and very short thermal increase and without damaging and modifying their chemical structure. The aim of this study was to investigate the possibility to condition the ceramic surfaces to improve their adhesion to the hard dental tissues. Despite further studies being necessary to fully confirm this hypothesis, both from the point of view of the mechanical properties of irradiated ceramic surfaces (micro-hardness, roughness) and the adhesion characteristics after ceramic sealing (wettability, shear bond strength, and micro-leakage), this preliminary study may be considered a demonstration of the hypothesis.

## 5. Conclusions

The use of a 1070 nm pulsed fiber laser represents a new interesting opportunity in the field of dentistry, thanks to the possibility of emission in ns pulse duration, limiting the collateral damage due to the overheating of the target, both tissues and materials. This preliminary study, performed on disilicate ceramic samples, showed the capacity of this device, with the proper parameters described before, to condition the dental porcelain, making it rougher without, by proper parameters, modifying its structure, and overheating and consequently damaging and destroying its surface layer.
